# Eosinophilic Myocarditis Treated with IL-5 Blockade: An Integrated Case Report and Literature Review

**DOI:** 10.3390/jcm14196829

**Published:** 2025-09-26

**Authors:** Hidenori Takahashi, Toru Awaya, Hiroki Nagamatsu, Yugo Satake, Ryutaro Hirose, Naoya Toba, Mio Toyama-Kousaka, Shinichiro Ota, Miwa Morikawa, Yuta Tajiri, Yoko Agemi, Natsuko Nakano, Masaharu Shinkai

**Affiliations:** 1 Department of Respiratory Medicine, Tokyo Shinagawa Hospital, 6-3-22, Higashi-Oi, Shinagawa-ku, Tokyo 140-8522, Japan; 2 Department of Cardiovascular Medicine, Tokyo Shinagawa Hospital, 6-3-22, Higashi-Oi, Shinagawa-ku, Tokyo 140-8522, Japan; toru0228@gmail.com (T.A.);; 3 Department of Cardiovascular Medicine, Toho University Ohashi Medical Center, 2-22-36, Ohashi Meguro-ku, Tokyo 153-8515, Japan; 4 Department of Respiratory Medicine, Yokohama Municipal Citizen’s Hospital, 1-1, Mitsuzawa-Nishimachi, Kanagawa-ku, Yokohama 221-0855, Kanagawa, Japan; 5 Department of Diagnostic Pathology Tokyo Shinagawa Hospital, 6-3-22, Higashi-Oi, Shinagawa-ku, Tokyo 140-8522, Japan

**Keywords:** eosinophilic myocarditis, eosinophilic granulomatosis with polyangiitis, hypereosinophilic syndrome, IL-5 blockade, mepolizumab, benralizumab, Charcot–Leyden crystals, case report, literature review

## Abstract

**Background/Objectives:** Eosinophilic myocarditis (EM) is a rare, life-threatening inflammatory cardiomyopathy driven by eosinophil cytotoxicity and extracellular trap formation. Interleukin-5 (IL-5) inhibition may disrupt this pathogenic cascade. We reviewed contemporary evidence on IL-5 blockade in EM and contextualized it with an illustrative case. **Methods:** We searched PubMed through May 2025 for reports of EM treated with mepolizumab or benralizumab. Inclusion criteria were consistent with prior cohorts: acute cardiac symptoms with biomarker elevation plus abnormalities on transthoracic echocardiography and/or cardiac magnetic resonance imaging (CMR), along with documented IL-5-targeted therapy. We extracted clinical, imaging, biopsy, treatment-timing, and outcome data and included one institutional case. **Results:** Twenty-one episodes were analyzed (median age, 45 years; 10 men). Underlying conditions included eosinophilic granulomatosis with polyangiitis (10 cases; 48%), hypereosinophilic syndrome (5 cases; 24%), drug reaction with eosinophilia and systemic symptoms (DRESS, 3 cases; 14%), and eosinophilic asthma (3 cases; 14%). Treatments involved mepolizumab in 17 cases (81%) and benralizumab in 4 (19%); 4 patients received “early-start” therapy within 14 days of EM diagnosis. Among the 11 episodes with reported left ventricular ejection fraction (LVEF) at baseline and follow-up, the median baseline LVEF was 40% (range, 30–62), with 10 of 11 (91%) <50%. On follow-up, all 11 patients improved: 4 normalized (≥50%) and 7 improved to 40–49%. CMR (n = 18) demonstrated late gadolinium enhancement in 14 cases (78%), edema in 9 (50%), and intracardiac thrombus in 4 (22%). Endomyocardial biopsy confirmed eosinophilic infiltration in 13 of 15 cases (87%). Outcomes included one death (fulminant DRESS), one recovery following veno-arterial extracorporeal membrane oxygenation, and one successful heart transplantation. **Illustrative case:** A 24-year-old man on a steroid taper received mepolizumab 300 mg on Day 4. His LVEF improved from 47% to 59% by Day 15, accompanied by biomarker decline and successful steroid tapering. **Conclusions:** Across published cases and our institutional experience, IL-5–targeted therapy appears safe, steroid-sparing, and associated with rapid ventricular recovery, particularly when initiated early. Although limited, these findings support the need for prospective trials to define the optimal agent, dosing, timing, and integration with standard immunosuppression and anticoagulation.

## 1. Introduction

### 1.1. Clinical Course and Pathophysiology of Eosinophilic Myocarditis

Eosinophilic myocarditis (EM) is an uncommon but potentially fatal form of inflammatory cardiomyopathy. Patients typically present acutely with chest pain, dyspnea, palpitations, or malignant ventricular arrhythmias. In fulminant cases, rapid progression to cardiogenic shock may occur [1]. Without timely diagnosis and treatment, the disease can be lethal: approximately 10% of patients die during hospitalization, and nearly 30% do not survive beyond three years [2]. Long-term outcomes are often compromised by myocardial fibrosis, persistent ventricular dysfunction, and thromboembolic complications [3].

Histologically, EM is defined by dense eosinophil infiltration within the myocardium, often accompanied by myocyte necrosis and interstitial edema [4]. Activated eosinophils release cytotoxic granule proteins—notably major basic protein and eosinophil cationic protein—that disrupt sarcolemmal membranes and amplify secondary inflammation. Additionally, eosinophil extracellular trap cell death (EETosis) contributes to sustained, and sometimes steroid-resistant, inflammatory cytokine release [5,6,7,8,9]. Uncontrolled injury promotes microthrombus formation [10,11] and, over weeks to months, can evolve into diffuse endomyocardial fibrosis (Löffler endocarditis), resulting in restrictive or dilated cardiomyopathy [1,12].

### 1.2. Etiologic Spectrum

The causes of EM are heterogeneous and can be broadly grouped into four categories [3]:

1. **Drug-induced or hypersensitivity reactions** (e.g., antibiotics, antiepileptics, nonsteroidal anti-inflammatory drugs, vaccines), including severe manifestations such as drug reaction with eosinophilia and systemic symptoms (DRESS) and Stevens–Johnson syndrome [13,14,15];
2. **Immune-mediated disorders**, chiefly eosinophilic granulomatosis with polyangiitis (EGPA) and hypereosinophilic syndromes (HES) [16,17,18,19,20,21,22,23,24,25,26,27,28]. Cardiac involvement is a leading cause of EGPA-related mortality; estimates vary, but up to approximately 30% of patients are affected, with cardiac disease accounting for nearly half of deaths [29,30]. Other eosinophilic/atopic conditions, such as allergic bronchopulmonary mycosis (ABPM) and eosinophilic asthma, are recognized causes of peripheral eosinophilia and, albeit rarely, can involve the myocardium [31,32,33,34];
3. **Parasitic, viral, and fungal infections** (e.g., toxocariasis, trichinosis, strongyloidiasis, helminths [35]; selected viral [22] and fungal pathogens [34]);
4. **Myeloproliferative and idiopathic entities**, including hematologic malignancy-associated eosinophilia [36].

This broad spectrum underlies the clinical variability that complicates early recognition and management.

### 1.3. Diagnostic Work-Up

**Laboratory and electrocardiogram (ECG).** Peripheral eosinophilia (>0.5 × 10^9^ cells/L) is observed in approximately 70% of cases but may be absent early [3]. Cardiac biomarkers (troponin, B-type natriuretic peptide [BNP]) are usually elevated. ECG often reveals nonspecific ST–T abnormalities or atrioventricular block [2,3].

**Echocardiography.** Transthoracic echocardiography (TTE) typically reveals global or regional left ventricular (LV) dysfunction that appears disproportionate to symptom duration, transient concentric wall thickening from edema or infiltration, apical/mural thrombus with spontaneous echo contrast, a restrictive filling pattern, reduced global longitudinal strain (GLS), and sometimes right ventricular (RV) involvement or a small pericardial effusion [3,12] GLS impairment often precedes declines in LV ejection fraction (LVEF), making it a sensitive early marker of ventricular dysfunction [37].

**Cardiac Magnetic Resonance (CMR).** CMR typically demonstrates nonischemic subendocardial or mid-wall late gadolinium enhancement (LGE), along with T2-weighted or T2-mapping abnormalities indicative of myocardial edema [38]. According to the 2018 revised Lake Louise Criteria, at least one T1-based and one T2-based abnormality are required to support active myocarditis [3,38,39,40,41]. In EM, subendocardial LGE has been linked to a higher prevalence of intracardiac thrombus and a greater incidence of major adverse cardiac events compared with mid-wall or subepicardial LGE patterns [42].

**Endomyocardial Biopsy (EMB).** When feasible, EMB remains the diagnostic gold standard for defining histotype and excluding mimics [43]. It can confirm eosinophil-rich infiltrates with or without necrosis and is particularly valuable in steroid-refractory or fulminant presentations [44,45,46].

### 1.4. Current Therapeutics and Unmet Needs

High-dose corticosteroids (e.g., intravenous methylprednisolone pulses followed by 0.5–1.0 mg/kg/day of prednisone equivalent) remain the first-line therapy and typically achieve rapid eosinophil suppression [1]. However, relapse is common during tapering, and cumulative toxicity limits long-term use [23]. Conventional immunosuppressants (e.g., cyclophosphamide, azathioprine, mycophenolate, rituximab) have been used in refractory disease, but supporting evidence is limited to small case series [4,16,18,19,28]. Anticoagulation is indicated when mural thrombus or extensive endocardial injury is present, given the prothrombotic milieu created by extracellular traps and immunothrombosis [3,10,11]. Despite contemporary care, reported in-hospital mortality remains 10–20% and is higher in hypersensitivity or necrotizing variants; long-term prognosis worsens once advanced fibrosis develops [2,3].

**Guideline note**: The 2024 ACC Expert Consensus Pathway recommends immunosuppression—typically high-dose corticosteroids—once infection has been excluded but does not provide guidance on IL-5–targeted biologics [38]. In contrast, the 2023 Japanese Circulation Society (JCS) guideline also advocates early corticosteroid use in EM but explicitly notes that mepolizumab may be considered in recurrent cases [43].

### 1.5. Rationale for IL-5—Targeted Therapy

Interleukin-5 (IL-5) regulates eosinophil proliferation, activation, trafficking, and survival. Two biologics disrupt this pathway: mepolizumab, which neutralizes circulating IL-5, and benralizumab, which blocks IL-5Rα. Both therefore inhibit IL-5 signaling; in addition, benralizumab is an afucosylated IgG1 that engages FcγRIIIa on natural killer cells to trigger antibody-dependent cellular cytotoxicity (ADCC), inducing apoptosis of eosinophils and basophils and yielding near-complete depletion of eosinophils in blood and tissue [47]. Both agents are approved for severe eosinophilic asthma; mepolizumab 300 mg subcutaneously every 4 weeks is also standard for relapsing/refractory EGPA based on the MIRRA phase 3 trial [48]. More recently, the 2024 MANDARA trial demonstrated noninferiority of benralizumab to mepolizumab for inducing remission in relapsing/refractory EGPA, with deeper eosinophil depletion and higher steroid-free rates [49]. Translational logic and accumulating case-level evidence suggest that IL-5 blockade may interrupt eosinophil-driven myocardial injury in EM, particularly when initiated early [5,6,27].

### 1.6. Knowledge Gap Highlighted by EGPA Cardiac Cohorts

A recent multicenter study proposed the LATE-EAST score to differentiate EGPA-associated EM (EGPA-EM) from EGPA-related chronic inflammatory cardiomyopathy. EGPA-EM was characterized by higher eosinophil counts, lower antineutrophil cytoplasmic antibodies (ANCA) positivity, more severe LV dysfunction, and higher mortality, underscoring the need for prospective trials of IL-5–targeted therapy in this subgroup [30,50].

## 2. Aim and Methods of the Present Review

**Aim.** To synthesize biologic-era evidence regarding IL-5 inhibition in EM and contextualize it with an illustrative case.

**Search Strategy.** We searched PubMed from inception through May 2025 using the terms *eosinophilic myocarditis*, *EGPA*, *HES*, *mepolizumab*, and *benralizumab*. Inclusion criteria followed those of Liu et al.: clinical evidence of EM (acute chest pain or heart-failure symptoms with elevated cardiac biomarkers and echocardiographic or CMR abnormalities) plus documented IL-5 inhibitor therapy [50]. Two reviewers independently screened and abstracted data; discrepancies were resolved by consensus. Twenty published cases met eligibility criteria, and we added one institutional case, yielding 21 analyzable episodes. This narrative review summarizes current understanding of EM pathophysiology, presents an additional case, and critically evaluates the merging clinical experience with IL-5 blockade. Our objective was to assess whether early adjunctive IL-5 inhibition mitigates myocardial injury, facilitates steroid withdrawal, and improves long-term cardiac outcomes.

## 3. Case Presentation

### 3.1. Clinical History

A 24-year-old Japanese man with childhood-onset asthma and atopic dermatitis presented unscheduled to the respiratory outpatient clinic (Day 1 of admission) with several days of sudden-onset pleuritic chest pain and dyspnea. The patient was tapering oral prednisolone prescribed for chronic eosinophilic pneumonia (CEP). He was a lifelong non-smoker, denied alcohol use, and reported aeroallergies to cedar pollen, dust mites, and house dust. Family history was negative for eosinophilia, vasculitis, or autoimmune disease.

### 3.2. Past Respiratory Course

**Figure 1:**
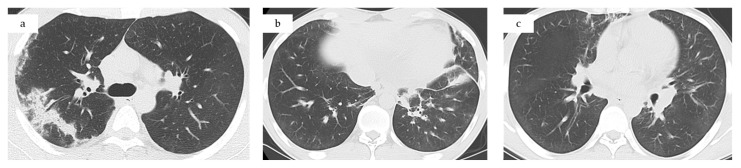
Chest CT at (**a**) initial presentation, (**b**) relapse of chronic eosinophilic pneumonia, and (**c**) current admission, all showing bilateral ground-glass opacities and patchy consolidations.

**Figure 2:**
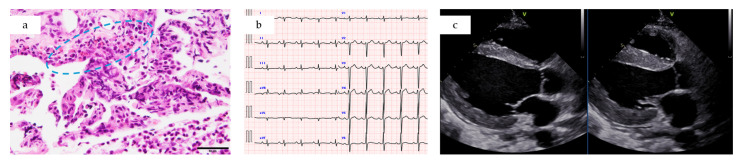
(**a**) Lung biopsy at the time of CEP diagnosis (Hematoxylin and Eosin [H&E] stain) showing marked eosinophilic infiltration (400× magnification). Areas of eosinophil accumulation are highlighted by the blue circle. Scale bar = 50 μm. (**b**) Electrocardiogram on current admission demonstrating nonspecific ST-T changes. (**c**) Transthoracic echocardiography on current admission in the parasternal long-axis view, showing end-diastolic (**left**) and end-systolic (**right**) frames with diffuse hypokinesis, myocardial edema, and a small pericardial effusion.

Two years and nine months earlier, a routine chest radiograph demonstrated bilateral pulmonary infiltrates (Figure 1a). Peripheral blood revealed marked eosinophilia (2450 cells/μL), bronchoalveolar lavage contained 81% eosinophils, and transbronchial biopsy confirmed CEP (Figure 2a). Oral prednisolone 30 mg/day was initiated, resulting in rapid clinical and radiographic improvement. The disease remained stable until one year before admission, when relapse occurred during tapering at 2.5 mg/day (Figure 1b). Prednisolone was re-escalated to 30 mg/day, leading to symptom resolution and improvement in radiographic findings. Steroid tapering was resumed after stabilization.

Six months before admission, the eosinophil count had decreased to 260 cells/μL while on prednisolone 5 mg/day. The dose was reduced and maintained at 3 mg/day four months before admission. Two months later, routine blood work revealed rising eosinophils (1480 cells/μL), though the patient remained asymptomatic. Prednisolone was continued at 3 mg/day until the current episode.

### 3.3. Initial Evaluation

On arrival the patient was afebrile (37.3 °C), hemodynamically stable (blood pressure 100/61 mmHg; heart rate 83 bpm), and oxygenated adequately (SpO_2_ 96% on ambient air). Physical examination was unremarkable; no murmurs, friction rubs, crackles, peripheral edema, or rash were noted.

**Figure 3:**
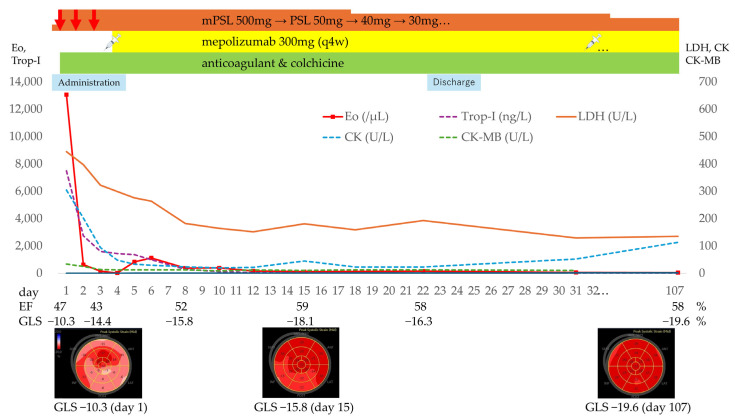
Clinical course depicting changes in eosinophil count and cardiac biomarkers, treatment regimen (mPSL, PSL, mepolizumab, anticoagulant, colchicine), and the patient’s discharge on Day 24. mPSL = methylprednisolone; PSL = prednisolone; Eos = eosinophil count per microliter; Trop-I = troponin I; EF = ejection fraction; GLS = global longitudinal strain.

- **ECG:** nonspecific ST-T changes (Figure 2b).
- **Transthoracic echocardiography:** diffuse hypokinesis, LVEF 47%, severely depressed myocardial GLS −10.3% (Figure 2c and Figure 3).
- **Laboratory findings**: white blood cell count 21,500/μL (eosinophils 13,060/μL), Creatine kinase [CK] 304 U/L, Creatine kinase–MB isoenzyme [CK-MB] 33.6 U/L, high-sensitivity troponin-I 7496 ng/L, BNP 145.9 pg/mL, fibrinogen/fibrin degradation products 4.2 µg/mL, D-dimer 1.8 µg/mL, serum IgE 1402 U/mL and Thymus and activation-regulated chemokine [TARC, also called CCL17] 1372 pg/mL (reference range: 0–450). C-reactive protein was 0.79 mg/dL, IL-6 mildly elevated at 15 pg/mL (reference range: 0–7.9) (Figure 3).
- **Computed tomography (CT) chest/abdominal:** recurrent bilateral ground-glass opacities and patchy consolidations without pleural effusion or lymphadenopathy, consistent with relapsed CEP. Abdominal and pelvic organs were unremarkable (Figure 1c).
- **Fractional exhaled nitric oxide (FeNO):** 197 ppb on Day 2 (previously 92 ppb four months earlier).

The combination of acute chest pain, cardiac enzyme elevation (CK-MB and troponin I), marked eosinophilia, and ventricular dysfunction was strongly suggestive of fulminant EM.

### 3.4. Diagnostic Work-Up

**Figure 4:**
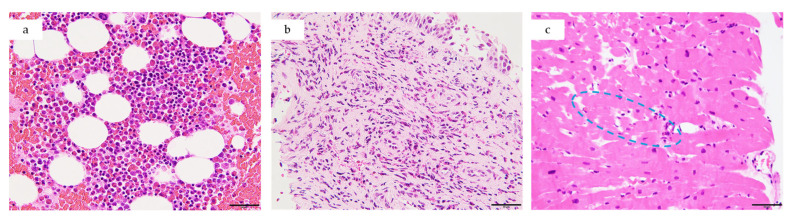
(**a**) Bone marrow, (**b**) sinonasal mucosa, and (**c**) myocardial tissue, all H&E-stained sections, demonstrating eosinophilic tissue infiltration. Blue circle highlights eosinophils infiltrating the myocardium, a finding not normally observed. All panels at 400× magnification. Scale bar = 50 μm.

Because of concerns regarding potential hemodynamic deterioration, EMB was deferred until stabilization. On Day 1, bone marrow aspirate revealed hypercellularity with approximately 30–40% nucleated eosinophil cells, no increase in blasts, and no features of myeloproliferative neoplasm (Figure 4a).

On Day 3, while receiving intravenous pulse corticosteroids, endoscopic sinonasal mucosa biopsy revealed dense eosinophilic infiltration (up to 80 eosinophils/high-power field) (Figure 4b). A right ventricular EMB performed the same day demonstrated myocardial edema with scattered eosinophils (1–3/high-power field) and lymphocytes between cardiomyocytes (Figure 4c). Persistence of eosinophils despite corticosteroid treatment supported the diagnosis of EM.

Right heart catheterization revealed a pulmonary capillary wedge pressure of 19/26/15 mmHg, pulmonary artery pressure of 28/12/19 mmHg, RV pressure of 38/0/4 mmHg, right atrial pressure of 5/4/2 mmHg, and cardiac output averaging 10.4 L/min. Cardiac catheterization and coronary angiography showed no stenosis.

**Figure 5:**
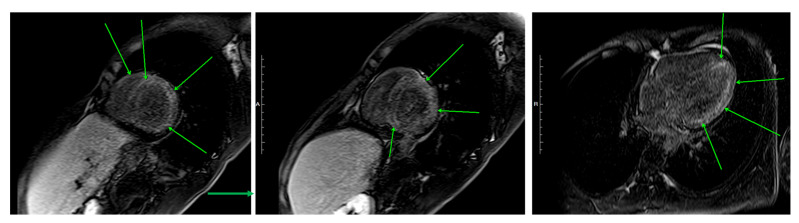
Cardiac MRI. Green arrows indicate areas of early gadolinium enhancement, reflecting myocardial injury.

Cardiac magnetic resonance imaging (MRI) on Day 8 (Figure 5) demonstrated mild concentric hypertrophy, globally reduced contractility, small circumferential pericardial effusion. Early gadolinium enhancement revealed diffuse, patchy uptake from the left ventricular apex to base, particularly in anterior/lateral walls (full-thickness), with relative septal sparing and a “sandwich pattern” (involving both the RV and LV subendocardium). Delayed enhancement images were technically inconclusive due to null-point inversion artifacts, but T2-weighted imaging confirmed diffuse myocardial hyperintensity consistent with edema. These findings, particularly subendocardial involvement, were atypical for other myocarditis types and highly suggestive of EM [51].

Serologic testing was negative for parasites, viruses (including SARS-CoV-2), and interferon-γ release assay. Autoimmune serologies, including PR3-ANCA and MPO-ANCA, were unremarkable. The FIP1L1–PDGFRA fusion transcript was not detected.

The patient fulfilled the 2022 ACR/EULAR classification criteria for EGPA [52].

### 3.5. Treatment

High-dose intravenous methylprednisolone (500 mg/day × 3 days) was initiated, followed by oral prednisolone 50 mg/day (approximately 0.8 mg/kg) (Figure 3). Concurrent therapy included unfractionated heparin (10,000 U/day × 7 days), which was then switched to edoxaban (60 mg/day for 2 weeks, tapered to 30 mg/day for one month, then 15 mg/day for six weeks) and discontinued after serial negative D-dimers, for a total anticoagulation period of approximately 3 months. Colchicine was introduced at 1.0 mg/day for 3 days, continued at 0.5 mg/day for anti-inflammatory/antifibrotic effect.

Despite eosinophil reduction and improvement in pulmonary infiltrates, cardiac function declined (LVEF 43%, GLS −14.4%) on Day 3. After multidisciplinary review, subcutaneous mepolizumab 300 mg was administered on Day 4. Thereafter, eosinophils remained <1000 cells/μL, troponin levels steadily decreased, and echocardiography showed rapid recovery: LVEF 52% by Day 8 and normalization to 59% (GLS −18.1%) by Day 15, with only a small residual pericardial effusion (Figure 3).

### 3.6. Clinical Course and Follow-Up

The patient remained hemodynamically stable throughout hospitalization, without arrhythmias or thromboembolism. Prednisolone was tapered cautiously during admission under serial monitoring of eosinophil counts, cardiac biomarkers, and echocardiographic parameters. The patient was discharged on Day 24 with the following home medications: prednisolone 30 mg/day, mepolizumab 300 mg every four weeks, colchicine 0.5 mg/day, and bisoprolol 0.625 mg/day. At discharge, eosinophils were 160 cells/μL, CK 23 U/L, CK-MB 12.8 U/L, troponin I 22.3 ng/L, and BNP 59.5 pg/mL.

Holter monitoring on Day 17 showed no arrhythmias or ST-T changes. No cutaneous, hepatic, renal, or other extracardiac involvement emerged. FeNO decreased to 36 ppb one week post-discharge. At outpatient follow-up, prednisolone was reduced gradually to 25 mg, 20 mg, 15 mg, and subsequently to 12.5 mg/day. Three-month follow-up echocardiography revealed LVEF 58% with near-normal GLS (−19.6%) and resolution of pericardial effusion (Figure 3). Biomarkers normalized (eosinophils 47 cells/μL, troponin I 9.4 ng/L, BNP 18.5 pg/mL, IgE 50 U/mL, TARC/CCL17 132 pg/mL, and D-dimer 0.2 μg/mL). Prednisolone was reduced to 10 mg/day on day 107.

At nine months, the patient was asymptomatic, working full-time, and exercising moderately on maintenance mepolizumab 300 mg every four weeks and prednisolone 6 mg/day. By 17 months, prednisolone was tapered to 4 mg/day. At 19 months, the patient remained clinically stable and relapse-free.

### 3.7. Longitudinal Biomarker Evaluation in This Case

On admission, a type 2–high, IL-5–anchored profile was present with eosinophilia, high FeNO (nitric oxide generated by the airway epithelium and, measured to assess eosinophilic airway inflammation and aid in diagnosis and management in asthma), and increased IgE and TARC (a C-C motif chemokine receptor 4 ligand chemokine indexing Th2 cell trafficking). During treatment with corticosteroids and mepolizumab, contraction of the IL-5/eosinophil axis was documented; FeNO and TARC declined in parallel as overall type 2 activity receded and were interpreted as nonspecific indicators of type 2 inflammation, whereas total IgE decreased over follow-up and was treated as a background marker of atopy and type 2 immune skewing [53,54,55]. These biomarkers were used as adjuncts in the integrated assessment of disease activity; myocardial activity was assessed in conjunction with cardiac biomarkers (troponin I, CK, CK-MB, and BNP) and imaging (echocardiography/CMR).

## 4. Literature Synthesis

### 4.1. Cohort Characteristics

We identified 21 cases of EM treated with an IL-5-directed biologic, including 20 previously published cases and the present case [13,14,15,16,17,18,20,22,23,24,25,26,27,28,32,33,34,50]. The median age was 45 years (range: 8–72 years), and sex distribution was balanced (10 men, 11 women) (Table 1). Eight cases originated in the United States, four in Japan, and three in Australia. Underlying conditions included EGPA in 10 cases (48%), of which 7 were ANCA–negative; HES in 5 cases (24%); DRESS in 3 cases (14%); and eosinophilic asthma or allergic bronchopulmonary mycosis in 3 cases (14%). The median peripheral-blood eosinophil count at presentation was 4720 cells/μL (range: 270–25,530). Extracardiac involvement most often affected the airways, with asthma present in 13 cases (62%). Cutaneous involvement was reported in 6 cases (29%), and paranasal sinus or pulmonary disease in 4 cases each (19%).

### 4.2. Cardiac Presentation

#### 4.2.1. Transthoracic Echocardiography (TTE)

TTE was performed in all 21 cases. Baseline LVEF was explicitly reported in 11, with a median of 40% (range: 30–62). LVEF was reduced (<50%) in 10 of 11 cases (91%). On follow-up, all patients demonstrated improvement: 4 patients achieved ≥50% and 7 improved to 40–49%. Global wall-motion abnormalities and diastolic restriction resolved in most cases. Pericardial effusion was present in 10 cases (48%), persisting in 2 cases and resolving in the remainder.

#### 4.2.2. Cardiac Magnetic Resonance Imaging (CMR)

CMR was performed in 18 of 21 cases (86%). LGE was observed in 14 (78%), most commonly with a subendocardial pattern (n = 10), whereas only one case demonstrated subepicardial distribution. Myocardial edema, reflected by high signal intensity on T2-weighted imaging or elevated T2 mapping, was detected in 9 cases (50%). Intracardiac thrombus was identified in 4 cases (22%). Follow-up CMR was available in 10 patients. LGE findings showed complete resolution in 3, partial regression in 5, and were not reported in 2; myocardial edema resolved or diminished in 5 cases.

#### 4.2.3. Endomyocardial Biopsy

Endomyocardial biopsy was performed in 15 cases (71%), with eosinophilic infiltration confirmed in 13 (87%). Myocyte necrosis was observed in 4 severe episodes. Histopathology revealed mural thrombus in 3 cases and concomitant myocardial fibrosis in 2. In one case, a follow-up endomyocardial biopsy after IL-5-directed therapy demonstrated resolution of eosinophilic infiltration with residual mild myocyte hypertrophy [32].

### 4.3. Use of IL-5 Inhibitors

**Agents:** Mepolizumab was administered in 17 of 21 cases (81%), and benralizumab in 4 cases (19%). No significant differences in age, sex, or geographic distribution were observed between treatment groups. All patients who received benralizumab had underlying EGPA or eosinophilic asthma (Table 1).

**Dosing:** Mepolizumab was delivered subcutaneously at 300 mg every 4 weeks in 12 of 17 patients (71%). In one case of DRESS-associated myocarditis, the dose was escalated to 500 mg following relapse, suggesting a potential dose-dependent effect [13]. Three patients received 100 mg, while two reports did not specify the dose. Benralizumab was consistently administered at 30 mg subcutaneously (days 0 and 28, then every 8 weeks) in all 4 cases.

**Timing of initiation**: IL-5 inhibitors were introduced in three clinical contexts: (1) initial therapy following EM diagnosis (n = 8), (2) relapse or worsening of EM (n = 10), and (3) steroid-sparing as maintenance therapy (n = 3). Among those who received IL-5 inhibitors early for initial treatment of newly diagnosed EM, the median interval from EM diagnosis to biologic initiation was 13 days (range: 3–42). Four patients received treatment within 14 days and are herein referred to as the “early-start” group.

- No cases of dose reduction, discontinuation, or switching of IL-5 inhibitors were reported during the treatment course in the reviewed literature.
- **Corticosteroids:** All 21 patients received high-dose corticosteroids at presentation.
- **Conventional immunosuppressants**: Eight patients (38%) also received immunosuppressants (rituximab, cyclophosphamide, azathioprine, or methotrexate), either before or concomitantly with IL-5 inhibition.

### 4.4. Clinical Outcomes

#### 4.4.1. Survival and Safety

Among 21 cases, one death was reported in a fulminant DRESS-associated case, where uncontrolled progression had already occurred before biologic initiation (Watanabe et al. [15], Table 1). One patient underwent heart transplantation for refractory cardiac failure (Sharma et al. [26], Table 1). All other patients were alive and clinically stable at the last follow-up. No serious adverse events attributable to IL-5 inhibitors were reported. Steroid-sparing was frequent, with durable remission in multiple reports.

#### 4.4.2. Therapeutic Impact

Improvement in LVEF or global systolic function was one of the most reported benefits of IL-5 inhibitor therapy, observed in 17 patients. Among 8 who received IL-5 inhibitors as initial therapy, 7 showed improved systolic function including all 4 in the early-start group. Symptom relief or clinical improvement was noted in 19 patients, steroid-sparing in 16. When restricting analysis to the initial-treatment subgroup (n = 8), these benefits were documented in 7 and 7 patients, respectively.

#### 4.4.3. Functional Recovery

Long-term prognosis was generally favorable. Among 21 patients, 9 with ≥12-month follow-up, had the most maintained remission with improved or stable LV function and successful steroid tapering (Table 1). Explicit reports of return to full daily or occupational activity were available in 9 cases, including the present case [18,22,23,24,25,32]. These comprised resumption of full-time work after ECMO recovery [25], complete symptom resolution within a week post-discharge with continued remission at 6 months [22], and sustained recovery without rehospitalization in all three patients from the Trovato series [24]. In the Higashitani case [18], independent ambulation was regained by 3 months with no subsequent relapse

## 5. Discussion

Accumulating case-level evidence suggests that IL-5 blockade—via mepolizumab or benralizumab—confers a remarkably consistent clinical benefit in EM. Across fulminant, relapsing, and chronic presentations, early biologic therapy has been observed to be associated with near-universal survival, rapid and durable recovery of ventricular function, successful corticosteroid tapering, and complete return to premorbid functional status. Although these findings derive from small series and individual reports, the concordant signal across diverse clinical contexts suggests that timely IL-5 inhibition may fundamentally alter the natural trajectory of this otherwise unpredictable disease [4].

### 5.1. Significance of Early IL-5 Inhibition in Eosinophilic Myocarditis

When introduced within approximately 14 days of symptom onset, IL-5 antagonists consistently achieved rapid hemodynamic stabilization, normalization of eosinophil counts, and full or near-complete recovery of LVEF. In contrast, delayed initiation—weeks to months into the disease course—was often followed by suboptimal outcomes, exemplified by one hypereosinophilic syndrome case that progressed to end-stage heart failure requiring cardiac transplantation [26] and a fulminant DRESS-associated case that proved fatal despite multidisciplinary therapy [15]. These observations parallel broader myocarditis literature: unchecked inflammatory injury leads to necrosis and fibrotic remodeling, processes that are increasingly irreversible once established. Accordingly, patients with more severe myocardial damage face poorer outcomes, and death or transplantation events occur predominantly within the first 90 days [56]. This underscores that early IL-5 blockade is critical for interrupting eosinophil-mediated injury before permanent scarring occurs [27].

### 5.2. Why Corticosteroids Are Necessary but Not Sufficient

High-dose systemic glucocorticoids are indispensable first-line therapy for eosinophilic myocarditis (EM); they rapidly suppress circulating eosinophils and can produce early improvement in left ventricular function. In a pooled analysis of 179 biopsy-proven cases [3], crude in-hospital mortality among non-EGPA/HES phenotypes appeared lower with steroid therapy than without (9.9% [10/101] vs. 65.7% [23/35]). Among survivors, the median LVEF at discharge was 57% for idiopathic/undefined EM, 60% for hypersensitivity EM, 40% for EGPA-related EM, and 49% for HES-related EM, possibly indicating greater residual systolic impairment in EGPA- and HES-associated disease despite steroid therapy.

Glucocorticoids rapidly decline in circulating eosinophils within 4–24 h and a slower resolution of tissue edema and infiltrates over days to weeks [57,58]. However, steroids have limited impact on EETosis and the downstream consequences of Charcot–Leyden crystal (CLC) deposition. During EETosis, activated eosinophils release DNA, granule proteins, and galectin-10, which crystallize into CLCs. These crystals are increasingly recognized as active drivers of persistent inflammation and fibrosis. CLCs are phagocytosed by macrophages, activate the NLRP3 inflammasome, and drive IL-1β release, thereby perpetuating fibro-inflammatory signaling [5,6,8,9]. Corticosteroids suppress cytokine transcription but do not dismantle existing CLCs nor fully inhibit NLRP3 activation, leaving patients vulnerable to steroid-refractory inflammation and progressive fibrosis [5,8].

### 5.3. Complementary Mechanisms of IL-5 Blockade

IL-5 antagonists provide upstream control that corticosteroids alone cannot. By rapidly depleting pathogenic eosinophils—mepolizumab via ligand neutralization, benralizumab via IL-5Rα-directed ADCC—these therapies suppress the principal source of EETosis [59], prevent new CLC formation [8], and interrupt the CLC-NLRP3-IL-1β cascade [9]. This mechanism likely explains the faster and more durable myocardial recovery observed with early biologic initiation [27,47]. Benralizumab, which nearly abolishes circulating blood and tissue eosinophils, may be particularly advantageous in fulminant EM, where even a small residual pool can perpetuate injury despite high-dose steroids. However, in cases with concurrent Th1/Th17-mediated vasculitis, IL-5 inhibition alone is insufficient, reinforcing the need for concomitant corticosteroids to suppress non-eosinophil inflammatory pathways and achieve disease control [21].

### 5.4. Mepolizumab and Benralizumab: Comparative Considerations

Randomized evidence remains limited to the MANDARA trial in refractory/relapsing EGPA, which demonstrates non-inferior remission rates at weeks 36 and 48 for benralizumab versus mepolizumab (58% vs. 56%), but with deeper eosinophil depletion (~30 vs. ~70 cells/µL) and a higher rate of steroid discontinuation (41% vs. 26%) [49]. Pharmacodynamically, mepolizumab selectively reduces IL-5–dependent inflammatory eosinophils, sparing some IL-5–independent resident eosinophils [60]. Benralizumab, in contrast, depletes nearly all IL-5Rα–expressing cells [47,61]. Inflammatory eosinophils flourish in IL-5–rich milieus and drive EETosis, CLC formation, and fibrosis in CLC–NLRP3–IL-1β cascade [5,8,9], whereas resident eosinophils—found in gut, adipose tissue, and epicardial fat—secrete IL-4/IL-13, promote M2 macrophage polarization, and facilitate tissue repair [62,63]. While full depletion may be desirable in fulminant EM, preservation of reparative eosinophils could support long-term remodeling. Pre-clinical studies suggest that total eosinophil knockout impairs ventricular healing after infarction [60], whereas IL-4–competent eosinophils limit scar formation [61]. Whether this nuance applies to EM remains unresolved. Importantly, the two-year follow-up data from MANDARA (ahead of print) showed that approximately one quarter of patients had baseline cardiac involvement, yet over the treatment period serious cardiac adverse events were infrequent (3/128, 2.3%), and no new cardiac safety signal emerged. Although patients with acute, organ-threatening cardiac disease were excluded, eosinophil depletion to near-zero levels was maintained in both continuous benralizumab and switch groups without apparent deterioration in cardiac status [64]. Benralizumab has since been approved for the maintenance of EGPA remission in the United States (September 2024), the European Union (November 2024), and Japan (December 2024). In contrast to its administration for severe asthma, where benralizumab is given every 4 weeks for the first three doses followed by dosing every 8 weeks, the approved regimen for EGPA is 30 mg subcutaneously every 4 weeks without interval extension [49]. With broader clinical availability, further data will likely accumulate to clarify its long-term efficacy and safety, including cardiac outcomes [59].

### 5.5. Dose Considerations: 300 mg vs. 100 mg Mepolizumab

In the largest real-world EGPA cohort (n = 203), mepolizumab 100 mg and 300 mg achieved similar global disease control, though only 10 patients had cardiac involvement and no dose-stratified cardiac outcomes were reported [20].

Separately, EM has been reported to develop in a patient with asthma and ANCA-negative EGPA while receiving mepolizumab 100 mg, suggesting that although this dose may control airway disease, it could be insufficient for suppressing fulminant, organ-threatening vasculitis or major cardiac or vascular involvement [21]. One of the reviewed cases [13] reported stabilization after dose escalation from 300 mg to 500 mg, suggesting a potential dose-dependent effect. Pharmacodynamically, the 300 mg dose achieves faster systemic exposure and more profound, sustained eosinophil suppression than 100 mg [61].

### 5.6. Imaging and Anticoagulation in EM: Thrombosis, Eosinophils, and IL-5 Blockade

EM frequently accompanies intracardiac and sometimes cerebral—thrombosis, making thrombus management a central component of care. For detection, CMR with LGE is the reference standard with high sensitivity and specificity. However, when MRI is not feasible, delayed-enhanced cardiac CT is a practical alternative, with contemporary meta-analyses supporting strong performance for left-heart thrombi (e.g., LAA and device-related thrombi) [65,66]. At the point of care, TTE can screen but has lower sensitivity and may miss laminated/small thrombi [65].

CMR studies demonstrate a close association between subendocardial injury and intracardiac thrombus formation [42]. Eosinophils contribute through multiple mechanisms: their granule proteins bind thrombomodulin, impairing endothelial anticoagulant function [67]; they directly activate coagulation factors; and eosinophil extracellular traps provide a prothrombotic scaffold that activates the contact pathway [3,10,11,68]. Accordingly, IL-5 inhibition, by rapidly depleting eosinophils, may complement anticoagulation in resolving intracardiac thrombi.

When the bleeding risk is acceptable, it is reasonable to initiate prophylactic parenteral anticoagulation (e.g., heparin) until brain MRI, cardiac MRI, and/or delayed-enhanced CT clarify the thrombus/embolism status [65]. Ongoing anticoagulation therapy should then be individualized based on imaging findings (thrombus presence/extent and degree of subendocardial injury), LVEF, and bleeding risk. While warfarin is traditionally used, direct oral anticoagulants are now widely employed for the prevention/treatment of intracardiac thrombi; selection should consider renal function, drug–drug interactions, and indication-specific evidence [69]. Regarding discontinuation, practical triggers include documented thrombus resolution on repeat imaging with recovery of LV function (for example, LVEF above approximately 35%) and no persistent apical akinesis, whereas factors supporting continuation include a protruding or mobile thrombus, prior embolic events, pro-inflammatory or hypercoagulable states, recurrent LV thrombus, and low bleeding risk. In persistent laminated/organized thrombus, discontinuation after shared decision-making may be reasonable [65]. Given that eosinophil extracellular trap–driven immunothrombosis and corticosteroid exposure may influence coagulation in EM [7,10,11,68,70], and that EM-specific data are scarce, duration and de-escalation should be guided by follow-up imaging and clinical recovery.

### 5.7. Colchicine and Myocarditis

Eosinophil granule proteins also promote fibrosis via IL-1β and transforming growth factor-β release [30]. Additionally, IL-1 directly contributes to myocardial injury and perpetuates a self-amplifying inflammatory loop in myocarditis [71]. Low-dose colchicine, a widely available microtubule inhibitor, blocks NLRP3 inflammasome assembly and attenuates IL-1β release. By dampening CLC-induced inflammasome activation, colchicine could theoretically mitigate ongoing fibrosis in EM [13,24,32]. In fact, a patient with EGPA-related EM experienced noticeable relief of recurrent chest pain with colchicine as initial therapy, suggesting its potential symptomatic benefit in the early phase of the disease [24]. Prospective studies are warranted to explore its role as an adjunct to IL-5 inhibition.

### 5.8. Limitations

The available evidence remains limited to small series and individual case reports, with heterogeneity in dosing, timing of therapy, and follow-up duration, raising the possibility of publication bias. Fatal cases may also be underreported. Disease-specific analyses (e.g., EGPA vs. HES vs. DRESS) are underpowered. Notably, cardiac involvement is more common in ANCA-negative EGPA [50,52], and strongly predicts adverse outcomes [29,50], yet robust evidence on differential steroid responsiveness by ANCA status is lacking, with prior reports yielding mixed results. Moreover, while small series suggest that mepolizumab may reduce ANCA titers in ANCA-positive EGPA, the clinical implications remain uncertain [72]. Given the rarity of EM and EGPA with cardiac involvement, adequately powered prospective studies are inherently challenging [30,64]. Registry-level data and controlled trials are therefore needed to clarify optimal timing, dose, duration, and potential combination strategies, including adjunctive immunomodulators or anticoagulation agents.

## 6. Conclusions

EM is a rare but potentially fatal form of inflammatory cardiomyopathy that requires early recognition and aggressive management. High-dose corticosteroids remain the cornerstone of therapy, but may not fully suppress eosinophil-mediated injury or prevent relapse. Our case and review of 21 reported cases suggest that early IL-5 pathway inhibition with mepolizumab or benralizumab accelerates ventricular recovery, enables corticosteroid tapering, and reduces recurrence risk, even in fulminant presentations. Mechanistic insights into eosinophil extracellular traps, CLC formation and NLRP3 inflammasome activation provide a strong biological rationale for IL-5 blockade in EM. Future prospective studies are essential to determine the optimal agent, dosing strategy, and timing of IL-5 inhibition, and to evaluate combination approaches with immunosuppressants and anticoagulation.

## Figures and Tables

**Table 1:** (**a**). Summary of 17 eosinophilic myocarditis episodes treated with mepolizumab. (**b**). Summary of four eosinophilic myocarditis episodes treated with benralizumab.

(**a**)										
**Author, Year**	**Age,** **Sex**	**Country**	**Etiological Background**	**Eos (/μL)**	**Extra-Cardiac Involvement**	**Echocardiographic Findings|Cardiac MRI Findings (→ After Treatment)**	**Cardiac Histopathology**	**Treatment**	**Timing of Mepo after EM—Indication**	**Mepolizumab Effect—Long Outcomes**
Song, 2017 [16]	60, M	USA	HES or ANCA-negative EGPA	7800	Sinus, asthma	EF 30%, large pericardial effusion, global hypokinesis → 35–40%|Diffuse endomyocardial infiltration	Eosinophilic infiltration with early thrombus; no vasculitis	Steroids → Aza → RTX → mepo (100 mg)	6 months—Treatment at relapse/worsening	Steroid-sparing, EF stabilization, clinical improvement—stable at 7 months
Kowtoniuk, 2018 [13]	45, F	USA	DRESS (caused by lamotrigine)	270	Skin	EF 30–34% → 60% (2 weeks)|Patchy mid-myocardial/subendocardial edema + LGE, improved	Dense eosinophilic infiltrates with necrosis, rare giant cells	Steroids → CPA → mepo (300 mg→500 mg)	6 weeks—Treatment at relapse/worsening	Steroid-sparing, clinical improvement—remission at 12 months
Truong, 2021 [14]	50 s, M	Australia	DRESS (caused by ciprofloxacin)	3800	Thyroid, skin	EF 33% → 65% (within 2 weeks)|Not reported	Mixed lymphohistiocytic + eosinophilic infiltrates, no necrosis	Steroids → mepo (300 mg) + CPA	Day 13—Initial therapy	Steroid-sparing, EF stabilization, clinical improvement—stable at 9 months
Huynh, 2022 [17]	20, M	Australia	HES	2200	Bone marrow	EF reduced → normalized (Day 19)|Atrial-predominant fibrosis	Eosinophilic infiltration	Steroids → mepo (300 mg) + CPA	Day 19—Treatment at relapse/worsening	Clinical improvement, EF stabilization—symptom-free at 6 months but persistent sinus arrest
Higashitani, 2022 [18]	46, F	Japan	ANCA-negative EGPA	3250	Asthma, mononeuritis multiplex	EF 41%, LV thickening, pericardial effusion → EF 48%|Diffuse edema, high ECV, mid-wall LGE; regressed	Eosinophilic infiltration, no vasculitis	Steroids → mepo (300 mg) + RTX	Day 40—Initial therapy	Steroid-sparing, EF improvement, clinical improvement—stable at 6 months
Ulu, 2023 [19]	17, F	Türkiye	ANCA-negative EGPA	1500	Skin, lung, bone marrow	Pericardial effusion 14 mm → resolved|Acute myocarditis with edema → resolved	Not performed	Steroids + IVIG + MTX → CPA → RTX→mepo (100 mg)	3 months—Treatment at relapse/worsening	Steroid-sparing, clinical improvement, EF stabilization –remission at 12 months
Wang, 2023 [20]	36, F	Taiwan	MPO-ANCA-positive EGPA	7140	Asthma, mononeuritis multiplex	EF reduced → normalized (12 months)|Diffuse mid-wall/endocardial LGE + edema → edema resolved, LGE ↓	Not described	Steroids → mepo (100 mg)	1 month—Treatment at relapse/worsening	Steroid-sparing, EF normalization, clinical improvement—remission at 12 months
Rao, 2023 [22]	20, M	USA	ANCA-negative EGPA	10,227	Sinus, asthma, lung	EF 40% → 45%|Diffuse edema + transmural LGE + mural thrombi → improved, residual LGE	Eosinophilic infiltration with thrombus	Steroids + mepo (300 mg)	Day 3—Initial therapy	EF improvement, clinical improvement—stable at 6 months
Panina, 2023 [23]	8, F	Latvia	ANCA-negative EGPA	25,530	Sinus, skin	EF normal; RVH → decreased|Diffuse subendocardial LGE + edema → improved, residual LGE	Not performed (bone marrow: hypercellularity with eosinophilia)	Steroids + mepo (dose not stated)	10 months—Steroid-sparing as maintenance therapy	EF improvement, clinical improvement—not stated
Trovato, 2024 [24]	34, F	USA	HES	5260	Asthma	EF 31% → 46%|Myopericarditis with mural thrombus + fibrosis	Eosinophilic infiltration	Steroids → mepo (300 mg)	10 months—Treatment at relapse/worsening	Steroid-sparing, EF improvement, clinical improvement—remission at 16 months
Trovato, 2024 [24]	65, M	USA	EGPA (ANCA not reported)	990	Asthma	EF n/a → normalized|Acute on chronic myocarditis with patchy LGE + edema (CMR EF 50%)	Not described	Steroids → mepo (300 mg)	shortly after diagnosis—Initial therapy	Steroid-sparing, EF stabilization, clinical improvement—remission at 12 months
Trovato, 2024 [24]	61, F	USA	HES	11,300	Asthma	EF 35% → 40–45%|Active inflammation with subendocardial fibrosis	Not described	Steroids → mepo (300 mg)	3 months—Steroid-sparing as maintenance therapy	Steroid-sparing, EF improvement, clinical improvement—stable at 3 months
Watanabe, 2024 [15]	30, F	Japan	DRESS (caused by phenobarbital)	ND	Liver, skin	EF diffusely impaired (exact value not reported), LVH, pericardial effusion|Not reported	Extensive eosinophilic + macrophage infiltration	Steroids → CPA → mepo (300 mg)	8 months—Treatment at relapse/worsening	Only transient improvement—fatal course
Brick, 2024 [25]	38, F	Australia	Idiopathic HES (iHES)	9700	ND	EF n/a (MRI 41%) → normalized after mechanical support|Biventricular thrombi + transmural LGE→both improved	Biventricular thrombus with transmural necrosis	Steroids → mepo (not stated)	Day 87—Steroid-sparing as maintenance therapy	Steroid-sparing, EF improvement, clinical improvement—stable at 3 months
Sharma, 2024 [26]	51, F	USA	Idiopathic HES (iHES)	4180	Asthma,	EF 41%, biventricular apical thickened and thrombi → EF 60% post-transplant|Diffuse subendocardial LGE	No inflammatory infiltrate, granuloma, or fibrosis	Steroids → mepo (300 mg)	6 weeks—Initial therapy	Reduced disease flares—stable at 3 months
Tartaglia, 2025 [34]	72, M	Italy	ABPM	880	Asthma, lung	EF normal → normal|basal anterior and inferior LGE → improved	Eosinophilic infiltration with mural thrombus	Steroids + mepo (300 mg)	8 months—Treatment at relapse/worsening	Steroid-sparing, EF improvement, clinical improvement—stable at 6 months
This case, 2025	24, M	Japan	ANCA-negative EGPA	13,060	Sinus, asthma, lung, bone marrow	EF 47%, myocardial edema (Figure 2c) → EF 59% (Day 15)|biventricular subendocardial enhancement (Figure 5)	Mixed lymphohistiocytic + eosinophilic infiltrates, no necrosis	Steroids → mepo (300 mg)	Day 4—Initial therapy	Steroid-sparing, EF improvement, clinical improvement—remission at 19 months
(**b**)										
**Author, Year**	**Age,** **Sex**	**Country**	**Etiological background**	**Eos (/μL)**	**Extra-Cardiac Involvement**	**Echocardiographic Findings|Cardiac MRI Findings (→ After Treatment)**	**Cardiac Histopathology**	**Treatment**	**Timing of Benr After EM—Indication**	**Benralizumab Effect—Long Outcomes**
Colantuono, 2020 [27]	19, M	Italy	ANCA-negative EGPA	13,470	Asthma, colitis, neuropathy, skin	EF n/a (MRI 40%) → 60% (2 months)|Diffuse subendocardial edema/fibrosis → edema resolved, fibrosis persisted (12 months)	Eosinophilic myocarditis with subendocardial fibrosis.	Steroids → benra 30 mg	6 weeks—Initial therapy	Steroid-sparing, EF improvement, clinical improvement—remission at 12 months
Kodaka, 2022 [33]	72, F	Japan	Eosinophilic asthma	940	Asthma	EF 41% → 48%|Not reported	Eosinophilic infiltration with interstitial fibrosis	Steroids → benra 30 mg	3 years after onset—Treatment at relapse/worsening	Steroid-sparing, EF improvement, clinical improvement—remission at 12 months
Belfeki, 2022 [28]	66, M	France	MPO-ANCA-positive EGPA	2500	Neuropathy, renal	EF normal → normal|Patchy subepicardial LGE → resolved	Not described.	Steroids + RTX → relapse → benra 30 mg	After 3rd maintenance RTX (≈18 M)—Treatment at relapse/worsening	Steroid-sparing, EF improvement, clinical improvement—stable at 30 months
Goyack, 2023 [32]	51, M	USA	Eosinophilic asthma	18,150	Asthma	EF 30–34% → 40–44%|Diffuse subendocardial + septal LGE → improved, residual septal enhancement	Eosinophil degranulation → follow-up biopsy: no eosinophils, mild myocyte hypertrophy	Steroids → benra 30 mg	Day 10—Initial therapy	Steroid-sparing, EF improvement, clinical improvement—stable at 9 months

EM = eosinophilic myocarditis; EGPA = eosinophilic granulomatosis with polyangiitis; HES = hypereosinophilic syndrome; iHES = idiopathic hypereosinophilic syndrome; DRESS = drug reaction with eosinophilia and systemic symptoms; ABPM = allergic bronchopulmonary mycosis; ANCA = antineutrophil cytoplasmic antibody; MPO-ANCA = myeloperoxidase-ANCA; EF = ejection fraction; LVEF = left ventricular ejection fraction; LV = left ventricle; LVH = left ventricular hypertrophy; RVH = right ventricular hypertrophy; LGE = late gadolinium enhancement; CMR = cardiac magnetic resonance imaging (MRI); TTE = transthoracic echocardiography; GLS = global longitudinal strain; ECV = extracellular volume; EMB = endomyocardial biopsy; Eos (/μL) = eosinophil count per microliter; mepo = mepolizumab; benra = benralizumab; Aza = azathioprine; CPA = cyclophosphamide; MTX = methotrexate; RTX = rituximab; IVIG = intravenous immunoglobulin; M = male; F = female.

## Data Availability

Data is contained within the article.

## Abbreviations

ABPM	Allergic bronchopulmonary mycosis
ACR/EULAR	American College of Rheumatology/European Alliance of Associations for Rheumatology
ADCC	Antibody-dependent cellular cytotoxicity
ANCA	Antineutrophil cytoplasmic antibody
AZA	Azathioprine
BNP	B-type natriuretic peptide
CEP	Chronic eosinophilic pneumonia
CK	Creatine kinase
CK-MB	Creatine kinase-MB isoenzyme
CLC	Charcot–Leyden crystal(s)
CMR	Cardiac magnetic resonance
CPA	Cyclophosphamide
CT	Computed tomography
DRESS	Drug reaction with eosinophilia and systemic symptoms
ECG	Electrocardiogram
ECMO	Extracorporeal membrane oxygenation
ECV	Extracellular volume
EGPA	Eosinophilic granulomatosis with polyangiitis
EGPA-EM	EGPA-associated eosinophilic myocarditis
EM	Eosinophilic myocarditis
EMB	Endomyocardial biopsy
EETosis	Eosinophil extracellular trap cell death
Eos	Eosinophil count per microliter
FeNO	Fractional exhaled nitric oxide
GLS	Global longitudinal strain
H&E	Hematoxylin and Eosin
HES	Hypereosinophilic syndrome
IgE	Immunoglobulin E
iHES	Idiopathic hypereosinophilic syndrome
IL	Interleukin
IL-1β	Interleukin-1 beta
IL-5	Interleukin-5
IVIG	Intravenous immunoglobulin
LGE	Late gadolinium enhancement
LV	Left ventricle
LVEF	Left ventricular ejection fraction
MANDARA	Phase 3 trial comparing benralizumab with mepolizumab in EGPA
MIRRA	Phase 3 trial of mepolizumab in relapsing/refractory EGPA
MMF	Mycophenolate mofetil
MPO-ANCA	Myeloperoxidase-ANCA
MTX	Methotrexate
MRI	Magnetic resonance imaging
NLRP3	NOD-like receptor family pyrin domain-containing 3
PR3-ANCA	Proteinase 3-ANCA
RTX	Rituximab
RV	Right ventricle
TARC/CCL17	Thymus and activation-regulated chemokine, also known as CC chemokine ligand 17
TTE	Transthoracic echocardiography
V-A ECMO	Veno-arterial extracorporeal membrane oxygenation
